# Erie County Medical Society

**Published:** 1885-04

**Authors:** Edward Clark

**Affiliations:** Secretary


### ERIE COUNTY MEDICAL SOCIETY.

### *Special Meeting, March 7, 1885.*

A special meeting of the Medical Society of the County of Erie was held in accordance with the following request:

BUFFALO, N. Y., March 4, 1885.

*Dr. J. B. Andrews, President of the Medical Society of the County of Erie:*

DEAR SIR—In accordance with a resolution adopted by the Buffalo Medical and Surgical Association, at its meeting on March 3, 1885, and in compliance with the by-laws of the Medical Society of the County of Erie, we the undersigned do respectfully request you to call a special meeting of the Medical Society of the County of Erie at as early a date as possible for the purpose of considering and approving a draft of a law regulating and restricting the practice of midwifery in Erie county by others than legally authorized physicians. The shortness of the legislative session makes it imperatively desirable to meet this week in order to present the draft to the Erie county members of the Senate and Assembly on Saturday or Sunday, and to finish, thus, the first steps taken by the Buffalo Medical and Surgical Association.

(Signed) M. HARTWIG,
E. E. STORCK,
F. W. BARTLETT,
JOHN HAUENSTEIN,
WILLIAM MEISBURGER.

The meeting was called to order by the President of the society, Dr. J. B. Andrews, and the following members were in attendance: Drs. Hopkins, Dorland, W. W. Potter, Diehl, Hartwig, Gumaer, Porter, Pryor, Hebenstreit, Mann, Thornton, Walsh, E. H. Long, B. G. Long, Marcley, Granger, Meisburger, Haberstro, Dambach, Wilson and Clark.

Dr. Hartwig presented and read a revised version of a bill to be presented to the legislature, entitled, "An act regulating and restricting the practice of midwifery in Erie county by others than legally authorized physicians."

After the reading of the document, Dr. Hopkins moved that the bill be taken up and considered, section by section, either for adoption or amendment, which was carried.

Dr. Hartwig read Sections I. and II. of the bill, both of which were adopted as read.

Section III. was read, and Dr. Hopkins said that he thought the place and time of meeting of the Examining Board should be stated, and moved to amend said section by inserting the place of meeting, which shall be the city of Buffalo, and that due notice of such meetings shall be publicly given. Dr. Hopkins' amendment was accepted by Dr. Hartwig.

Dr. Pryor moved to further amend said section by inserting the clause, "The fee for such examination be $10.00, and that the fees collected by said Examining Board be expended in defraying the expenses incurred by the Board."

Dr. Haberstro moved to further amend Section III. by making the examination fee $25.00, instead of $10.00, as recommended in the bill. Dr. Walsh endorsed Dr. Haberstro's proposed amendment, as did also Dr. Dambach.

A vote being taken on Dr. Haberstro's motion, it was lost.

Section III., as amended by Dr. Pryor, was then adopted.

Sections IV., V., VI. and VII. were then read and adopted.

Dr. Hartwig moved that the bill, as amended, which is as follows, be now adopted as a whole, which was carried:

"AN ACT REGULATING AND RESTRICTING THE PRACTICE OF MIDWIFERY IN ERIE COUNTY BY OTHERS THAN LEGALLY AUTHORIZED PHYSICIANS.

"*The People of the State of New York, represented in Senate and Assembly, do enact as follows:*

"SECTION I. On or before the first day of July, 1885, the County Judge of Erie county shall, by an order to be filed in the Erie county Clerk's office, appoint a Board of Examiners in Midwifery, to consist of five members, who shall have been licensed to practice physic and surgery in this State, and thereafter as often as any vacancy shall occur in said Board, said County Judge shall, by a like order, fill such vacancy.

"SEC. II. Immediately after the filing of said order, said Board shall organize, by the selection of one of its members as President, and of another as Secretary, and Treasurer, who shall hold their office for one year and be thereafter annually elected, and shall adopt, and have power to adopt and enforce such rules and regulations as are necessary to carry out the purposes and provisions of this act.

"SEC. III. Such Examiners shall meet in the city of Buffalo, on the first Tuesday of October and April of each year, and such other days as such Board may appoint, due notice of such meetings to be publicly given, and shall then examine all candidates of the age of 21 years and upwards, and possessed of a good moral character, who shall present themselves to be examined for license to practice midwifery in the county of Erie, and shall on receipt of $10, issue their certificate to any person so examined who shall be found by them to be qualified, which certificate shall set forth that said Board has found the person to whom it is issued qualified to practice midwifery, and shall be recorded by the Clerk of the county of Erie, in a book, to be kept by him for that purpose. All moneys coming into the treasury of this Board shall be used to defray the expenses of the Board.

"SEC. IV. Any person who has received and recorded such certificate, shall, thereupon, be designated a midwife, and authorized and entitled, within the county of Erie, to practice midwifery in cases of normal labor and in no others; but such persons shall not in any case of labor use instruments of any kind, nor assist labor by any artificial, forcible, or mechanical means, nor perform any version, nor attempt to remove adherent placenta, nor administer, prescribe, advise, or employ, any poisonous or dangerous drug, herb or medicine; nor attempt the treatment of disease, except when the attendance of a physician cannot be speedily procured; and in such case, such person shall at once, and in the most speedy way, procure the attendance of a physician.

"SEC. V. Said Board of Examiners shall have power, on proper cause shown, and after hearing the person holding their certificate, to recommend to the County Judge of Erie county the revocation of the same, and said Judge shall have power so to do.

"SEC. VI. Any person who shall practice, or without the attendance of a physician, where one can be procured, attend a case of midwifery, or obstetrics, within the county of Erie, after the 31st day of December, 1885, without being duly authorized so to do, under existing laws of the State, or without having received and recorded the certificate for by this act; and any person who shall violate any of the provisions of this act, shall be guilty of a misdemeanor, and, on conviction thereof, shall be fined not less than $50.00, nor more than $100, and shall forfeit any certificate theretofore granted under the provisions of this act.

"Sec. VII. This act shall take effect immediately."

Dr. Hopkins moved that the bill be presented to the Committee on Legislation, for their consideration, and by them be forwarded to the members of the legislature for this county, which was carried.

Dr. Hartwig offered the following resolution, and moved its adoption, which was carried unanimously:

Whereas, The Medical Society of the County of Erie, in consideration of the growing evil of self-constituted midwives in this county, which evil has caused unnecessary losses of mothers and children to the community, and,

Whereas, This society desires to provide the necessary help for the poorer classes, who are unable to employ a legally authorized physician, therefore,

*Resolved*, That a copy of the foregoing draft of a law, together with this resolution, be transmitted to the members of the legislature representing this county, with the request that they use all laudable means to secure the passage of the foregoing act.

Dr. Potter moved that a vote of thanks be tendered the committee having in charge the consideration of a bill providing for the licensing of midwives, and that, as their duties are completed, the committee be now discharged. Carried.

There being no other business before the meeting, the society adjourned.

Edward Clark, *Secretary.*

